# Sex Differences in Academic Productivity Across Academic Ranks and Specialties in Academic Medicine: A Systematic Review and Meta-analysis

**DOI:** 10.1001/jamanetworkopen.2021.12404

**Published:** 2021-06-29

**Authors:** Giang L. Ha, Eric J. Lehrer, Ming Wang, Emma Holliday, Reshma Jagsi, Nicholas G. Zaorsky

**Affiliations:** 1 Department of Radiation Oncology, Penn State Cancer Institute, Hershey, Pennsylvania; 2 Department of Radiation Oncology, Icahn School of Medicine at Mount Sinai, New York, New York; 3 Department of Public Health Sciences, Division of Biostatistics and Bioinformatics, Penn State Cancer Institute, Hershey, Pennsylvania; 4 Department of Radiation Oncology, The University of Texas MD Anderson Cancer Center, Houston; 5 Department of Radiation Oncology, University of Michigan, Ann Arbor; 6 Center for Bioethics and Social Sciences, University of Michigan, Ann Arbor

## Abstract

**Importance:**

Despite equal numbers of men and women entering medical school, women are underrepresented in the upper echelons of academic medicine and receive less compensation and research funding. Citation-related publication productivity metrics, such as the h-index, are increasingly used for hiring, salary, grants, retention, promotion, and tenure decisions. Exploring sex differences in these metrics across academic medicine provides deeper insight into why differences are observed in career outcomes.

**Objective:**

To systematically examine the available literature on sex differences in h-index of academic faculty physicians across all medical specialties and all levels of academic rank.

**Data Sources:**

Medical literature with the term *h-index* found in PubMed and published between January 1, 2009, and December 31, 2018, was used.

**Study Selection:**

A PICOS (Population, Intervention, Comparison, and Outcomes), PRISMA (Preferred Reporting Items for Systematic Reviews and Meta-analyses), and MOOSE (Meta-analysis of Observational Studies in Epidemiology) selection protocol was used to find observational studies that published h-indexes for faculty physicians that were stratified by sex. Studies were excluded if they were review articles, retracted, or unavailable online. Ultimately, 14 of 786 studies (1.78%) met the inclusion criteria.

**Data Extraction and Synthesis:**

Data from 9 studies across 16 specialties were examined using weighted random-effects meta-analyses. Five studies were excluded because of overlapping specialties with another study or because they were missing appropriate statistics for the meta-analysis. Four of these studies were included in qualitative synthesis to bring the total to 13 studies.

**Main Outcomes and Measures:**

The primary study outcome was the h-index.

**Results:**

The meta-analysis included 10 665 North American unique academic physicians across 9 different studies from the years 2009 to 2018. Of the 10 665 physicians, 2655 (24.89%) were women. Summary effect sizes for mean h-indexes of men and women and mean h-index difference between men and women were determined for all faculty physicians and at each academic rank. Overall, female faculty had lower h-indexes than male faculty (mean difference, −4.09; 95% CI, −5.44 to −2.73; *P* < .001). When adjusting for academic rank, female faculty still had lower h-indexes than male faculty at the ranks of assistant professor (mean difference, −1.3; 95% CI, −1.90 to −0.72; *P* < .001), associate professor (mean difference, −2.09; 95% CI, −3.40 to −0.78; *P* = .002), and professor (mean difference, −3.41; 95% CI, −6.24 to −0.58; *P* = .02).

**Conclusions and Relevance:**

In this systematic review and meta-analysis, women had lower h-indexes than men across most specialties and at all academic ranks, but it is unclear why these differences exist. These findings suggest that future investigation should be conducted regarding the causes of lower h-indexes in women and that interventions should be developed to provide a more equitable environment for all physicians regardless of sex.

## Key Points

**Question:**

What are the sex differences in citation-related publication productivity across academic ranks and specialties in academic medicine?

**Findings:**

This systematic review and meta-analysis found that women had lower publication productivity than men. When productivity was evaluated separately by specialty, women had lower productivity than men in all analyzed specialties except for plastic surgery; when productivity was organized by rank, women had lower productivity than men at the ranks of assistant professor, associate professor, and professor.

**Meaning:**

These findings suggest that future investigation should be conducted regarding the causes of women’s decreased citation-related publication productivity within the field and interventions should be developed to provide a more equitable environment for all physicians, regardless of sex.

## Teaser

This systematic review and meta-analysis examines the available literature on sex differences in h-index, a measure of citation-related publication productivity, of academic faculty physicians across all medical specialties and all levels of academic rank.

## Introduction

Sex inequality continues to be a major concern in academic medicine. In 2018, women made up 50.9% of applicants to US medical schools, 41.4% of full-time clinical faculty at US medical schools, and 35.8% of the US physician workforce.^1^ Furthermore, specialties vary widely in female representation.^1,2^ Women remain in the minority among those in leadership positions and positions of higher academic rank.^3,4,5,6,7,8^ Women in medicine also have lower salaries,^9,10^ number of publications,^4,11,12^ and research funding^13,14^ compared with their male counterparts.

Potential reasons for female underrepresentation in the upper echelons of academic medicine have generated much discussion.^15^ One major component of performance assessment is academic influence; in particular, academic influence impacts the selection of residents, hiring and promotion of faculty physicians, and receipt of research funding.^16,17,18,19,20,21^ The h-index was developed as a metric to quantify the amount and impact of one’s publications and is equivalent to the highest number (*h*), so that *h* of the individual’s total number of publications (*N_p_*) have at least *h* citations, and the remaining number of papers (*N_p_* – *h*) have *h* citations or fewer.^22^ The m-index (or m-quotient) is a common variation that divides the h-index by the number of years since an individual’s first publication. The m-index mitigates some of the inherent time bias of the h-index and allows for comparison of researchers in different stages of their career.^22^ Because it is easy to calculate, benchmark, and compare, the h-index has earned a prominent place in the performance evaluation of faculty physicians.^23^ Despite its purpose as an objective metric, the h-index neglects to account for bias and disparity inherent in several determinants of publication authorship.^6,13,14,19,24,25,26,27,28^ Although studies^24,29,30,31,32,33,34,35,36,37,38,39,40^ have examined sex differences in academic influence within individual specialties, a gap remains in the study of sex differences in academic influence across medicine as a whole.

The purpose of this study was to systematically synthesize and examine the available literature on sex differences regarding h- and m-indexes of academic physician faculty across a wide range of specialties and all levels of academic rank. We hypothesized that women would have lower publication productivity citation-related metrics than men, particularly within specialties that are historically male dominated.

## Methods

**Figure 1.**
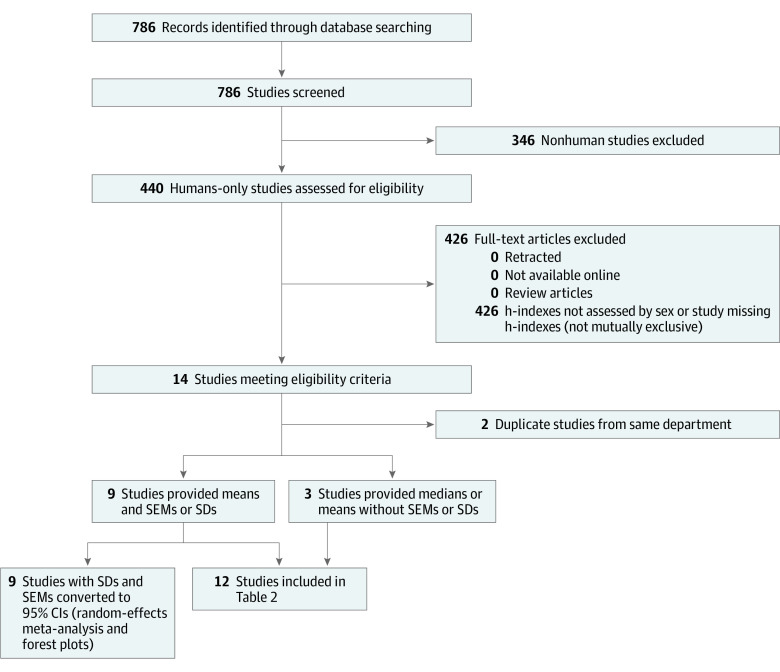
PRISMA Flow Diagram of Included Studies

The primary metric used for this study was the h-index. The secondary metric was the m-index. This meta-analysis was conducted and reported in accordance with the Preferred Reporting Items for Systematic Reviews and Meta-analyses (PRISMA) reporting guideline^41^ and the Meta-analysis of Observational Studies in Epidemiology (MOOSE) reporting guideline (Figure 1).^42^

### Data Sources and Searches

Medical literature, including observational studies, published in the English language from January 1, 2005, to December 31, 2018, was searched in multiple databases (Figure 1) using the term *h-index*. We aimed to identify all active clinical and nonclinical researchers. However, most studies were clinical and focused on academic medical subspecialties. We also used a long string search (Figure 1) to find relevant literature, but no additional results were produced.

### Study Selection

The inclusion criteria for the literature search were defined using the Population, Intervention, Control, Outcome, and Study Design (PICOS) approach (eTable in the Supplement).^43^ The population was composed of faculty in academic medicine with reported mean and/or median h-indexes. The studies must have not only reported h-indexes but also categorized these metrics by sex. On the basis of the inclusion criteria, 786 studies were screened by 1 of the investigators (G.L.H.). Of these, 440 humans-only studies were assessed for eligibility, and 426 were excluded if they were retracted, not available online, were review articles, did not include h-indexes and/or total sample sizes, and did not assess h-indexes by sex. Ultimately, 14 full-text articles met all the inclusion criteria, encompassing 16 different medical specialties. Some studies also stratified the h-index by academic rank: assistant professor, associate professor, professor, and chair.

### Data Extraction and Quality Assessment

Data in these articles were extracted by 1 author (G.L.H.), who was not involved in any of the studies. Discrepancies in values were resolved by discussion with 2 other investigators (N.G.Z. and E.J.L.). When multiple studies existed for the same specialty, the studies that were included were chosen based on whether they provided both means and either SEMs or SDs. If the studies provided the same descriptive statistics, studies were chosen based on whether they also assessed h-indexes by position and/or provided h-indexes across multiple specialties. This process eliminated 2 studies.^31,32^ However, 1 study^31^ reported m-indexes; m-indexes were included in this systematic review but not in the meta-analysis. Risk of bias was not assessed because the studies did not report any type of intervention.

### Data Synthesis and Analysis

Of the remaining 12 studies, 9^24,29,33,34,35,36,37,38,39^ reported the mean h-index and provided the data needed to calculate the SDs, SEMs, and 95% CIs. These 9 studies were included in the meta-analysis. Three studies^4,30,40^ did not directly provide numerical values for means, SDs, or SEMs but instead provided bar graphs that depicted these values. For those studies, we used Plot Digitizer, version 2.6.8 (Plot Digitizer) to approximate the values we needed to calculate SDs, SEMs, and 95% CIs. These 3 studies reported only median or mean h-indexes without SEMs or SDs. The authors of these studies were unsuccessfully contacted to supply missing data, and these studies^4,30,40^ were not included in the meta-analysis; however, they were still included in the subsequent systematic review. (Figure 1).

### Statistical Analysis

The data were analyzed using RStudio, version 1.1.383 (RStudio) and the Meta-Analysis Package for R (metafor), version 4.0.2 (Wolfgang Viechtbauer) to conduct the meta-analyses and heterogeneity tests. Meta-analyses were conducted on the difference in the h-index between men and women overall and by academic specialty. In particular, the random mixed-effects models with the restricted maximum likelihood approach were used for analysis.^44,45^ Hypothesis tests were 2-tailed, and the a priori level of significance was α = .05. Forest plots for overall findings and findings by academic specialty were generated to show the heterogeneity and significance of differences in h-indexes between men and women.

## Results

### Study Characteristics

The meta-analysis included a total of 10 665 unique North American academic physicians across 9 different studies from the years 2009 to 2018. A total of 2655 (24.89%) were women. Publication metrics were analyzed for anesthesiology,^33^ dermatology,^34^ general surgery,^33,35^ internal medicine,^33^ neurosurgery,^36^ obstetrics and gynecology,^33^ ophthalmology,^37^ orthopedic surgery,^29^ otolaryngology,^36^ pediatrics,^33^ plastic surgery,^36,38^ radiology,^33^ surgical oncology,^24^ and urology.^36,39^ Additional data on craniofacial surgery^40^ and radiation oncology^4,30^ were found but were not included in the meta-analysis because of the h-indexes being reported as median values or the reference not reporting an SEM or SD (Table 1). An additional study^31^ for neurosurgery was included in the systematic review for its analysis of m-indexes. All studies^4,24,29,30,33,34,35,36,37,39,40^ included in the systematic review assessed the sex of physicians through searching online faculty listings except for 2 studies,^31,38^ which did not state how sex was assessed. Furthermore, all studies attributed binary identity.

**Table 1.** Mean and Median h-Indexes by Specialty, Position, and Sex, 2009-2018

Specialty and position	Mean h-index (SEM)^a^		Publication	Discusses possible causes?	Discusses intervention?
	Women	Men			
Anesthesiology					
Professor	4.72 (1.12) (n = 20)	9.49 (0.76) (n = 82)	Pashkova et al,^33^ 2013	Yes; female anesthesiologists contribute more to clinical and educational domains	Yes; increase recruitment of research-avid female medical students
Overall	1.75 (0.24) (n = 198)	3.37 (0.24) (n = 447)		Women have only recently increased their numbers in this field	Increase mentorship for female trainees
				Lifestyle factors (eg, family care) decrease time devoted to academic work	Encourage women to be more proactive at seeking career opportunities early in training
Craniofacial surgery overall	6 (5.5)^b^^,^^c^ (n = 14)	12 (14)^b^^,^^c^ (n = 88)	Ruan et al,^40^ 2017	No	No
Dermatology					
Assistant professor	4.51 (n = 267)	6.51 (n = 199)	John et al,^34^ 2016	No	Yes; emphasize doing research early in residency training
Associate professor	10.70 (n = 98)	10.89 (n = 135)			
Professor	19.01 (n = 86)	25.02 (n = 182)			
Chair	16.68 (n = 23)	26.95 (n = 71)			
Overall	8.74 (0.42) (n = 474)	15.15 (0.61) (n = 587)			
General surgery					
Assistant professor	6.39 (0.91) (n = 23)	8.63 (0.80) (n = 51)	Mueller et al,^35^ 2017	No	No
Associate professor	11.3 (1.17) (n = 10)	14.93 (1.06) (n = 45)			
Professor	21.94 (3.07) (n = 18)	25.20 (1.65) (n = 59)			
Overall	6.31 (0.70) (n = 57)	11.73 (0.76) (n = 237)	Pashkova et al,^33^ 2013	No	No
Internal medicine overall	2.01 (0.37) (n = 149)	5.26 (0.63) (n = 197)	Pashkova et al,^33^ 2013	No	No
Neurosurgery overall	8.48 (1.63) (n = 19)	13.04 (0.95) (n = 171)	Eloy et al,^36^ 2013	Yes; mentorship opportunities are not as robust for women; educational and community service responsibilities are more often assigned to women, taking away from their academic time; women have increased family responsibilities	No
Obstetrics and gynecology overall	3.78 (0.39) (n = 153)	8.14 (0.81) (n = 148)	Pashkova et al,^33^ 2013	No	No
Ophthalmology					
Assistant professor	2.85 (0.21) (n = 271)	3.99 (0.25) (n = 348)	Lopez et al,^37^ 2014	Yes; familial obligations early in career for women lead to lower productivity early in career, higher productivity later in career when compared with men	No
Associate professor	8.00 (0.68) (n = 84)	8.36 (0.52) (n = 237)			
Professor	16.77 (1.41) (n = 50)	16.44 (0.73) (n = 361)			
Chair	18.31 (1.17) (n = 6)	15.64 (6.26) (n = 102)			
Overall	6.00 (0.38) (n = 419)	10.40 (0.34) (n = 1011)			
Orthopedic surgery					
Assistant professor	2.80 (0.30) (n = 133)	3.80 (0.16) (n = 843)	Bastian et al,^29^ 2017	No	No
Associate professor	6.50 (0.73) (n = 45)	8.60 (0.32) (n = 459)			
Professor	14.60 (1.61) (n = 19)	15.10 (0.53) (n = 442)			
Otolaryngology					
Assistant professor	3.82 (0.42) (n = 108)	4.32 (0.28) (n = 348)	Eloy et al,^36^ 2013	Yes; mentorship opportunities are not as robust for women; educational and community service responsibilities are more often assigned to women, taking away from their academic time; women have increased family responsibilities	No
Associate professor	7.78 (0.78) (n = 50)	8.99 (0.42) (n = 198)			
Professor	16.84 (1.49) (n = 31)	14.65 (0.71) (n = 227)			
Overall	7.34 (0.54) (n = 191)	9.27 (0.28) (n = 862)			
Pediatrics overall	2.78 (0.42) (n = 84)	4.57 (0.65) (n = 118)	Pashkova et al,^33^ 2013	No	No
Plastic surgery					
Assistant or associate professor	5.1 (0.46) (n = 67)	6.40 (0.33) (n = 254)	Paik et al,^38^ 2014	Yes; educational and community service responsibilities are more often assigned to women, taking away from their academic time	No
Professor	8.20 (1.72) (n = 6)	13.30 (0.89) (n = 101)			
Chair	14.30 (4.55) (n = 6)	11.90 (0.92) (n = 71)			
Overall	7.37 (1.33) (n = 15)	7.41 (0.65) (n = 93)	Eloy et al,^36^ 2013	Yes; mentorship opportunities are not as robust for women; educational and community service responsibilities are more often assigned to women, taking away from their academic time; women have increased family responsibilities	No
Radiology overall	4.52 (0.60) (n = 132)	7.25 (0.51) (n = 319)	Pashkova et al,^33^ 2013	No	No
Radiation oncology					
Assistant professor	3^b^ (n = 137)	4^b^ (n = 274)	Holliday et al,^4^ 2014	No	No
Associate professor	10^b^ (n = 54)	12^b^ (n = 115)			
Professor or chair	20.5^b^ (n = 293)	23^b^ (n = 738)			
Overall	6.4 (n = 234)	9.4 (n = 592)	Choi et al,^30^ 2009	Yes; there are much fewer women in academic radiation oncology, especially in higher ranks; there is a lack of female role models; familial obligations affect women more than men; subtle discrimination according to sex decreases the resources available for women	No
Surgical oncology					
Assistant professor	6.7 (0.53) (n = 111)	9.7 (0.58) (n = 75)	Nguyen et al,^24^ 2018	Yes; there are fewer available mentors and collaborators for women because the field is male dominated; female physicians may have authored papers under their maiden names, which may not be counted in the h-index; familial responsibilities affect female physicians more than male physicians; female physicians are more likely to focus on clinical excellence and teaching rather than research	No
Associate professor	15 (1.07) (n = 50)	20 (1.05) (n = 78)			
Professor	24 (2.17) (n = 36)	34 (1.75) (n = 107)			
Division chief	28 (3.05) (n = 13)	34 (2.43) (n = 55)			
Chair	25 (15.59) (n = 3)	51 (6.31) (n = 17)			
Overall	13 (0.75) (n = 213)	26 (1.04) (n = 331)			
Urology					
Assistant professor	5.63 (0.61) (n = 141)	7.49 (0.57) (n = 603)	Mayer et al,^39^ 2017	Yes; longer career duration for men; women are more likely to pursue clinical-educator track; women are pigeonholed (relegated to less academically productive subspecialties); women have more familial responsibilities than men	Yes; more same-sex mentors and better opposite-sex mentors to provide better mentorship for female urologists
Associate professor	10.63 (3.40) (n = 46)	14.19 (1.60) (n = 315)			
Professor	20.20 (1.64) (n = 25)	28.15 (0.81) (n = 441)			
Chair	27.33 (6.43) (n = 3)	31.54 (2.28) (n = 126)			
Overall	8.32 (1.60) (n = 27)	13.23 (0.80) (n = 239)	Eloy et al,^36^ 2013	Yes; mentorship opportunities are not as robust for women; educational and community service responsibilities are more often assigned to women, taking away from their academic time; women have increased family responsibilities	No

^a^ SEM only shown if directly provided or can be calculated from provided SD.

^b^ Median instead of mean.

^c^ Interquartile range instead of SEM.

### Publication Productivity and Sex

Table 1 lists the mean h-indexes of male and female academic physician faculty members by specialty and academic rank. There was consistently greater male representation across all specialties (except obstetrics and gynecology) and academic ranks. Table 1 also includes a summary of possible causes of observed sex differences and interventions proposed in the included studies.

**Figure 2.**
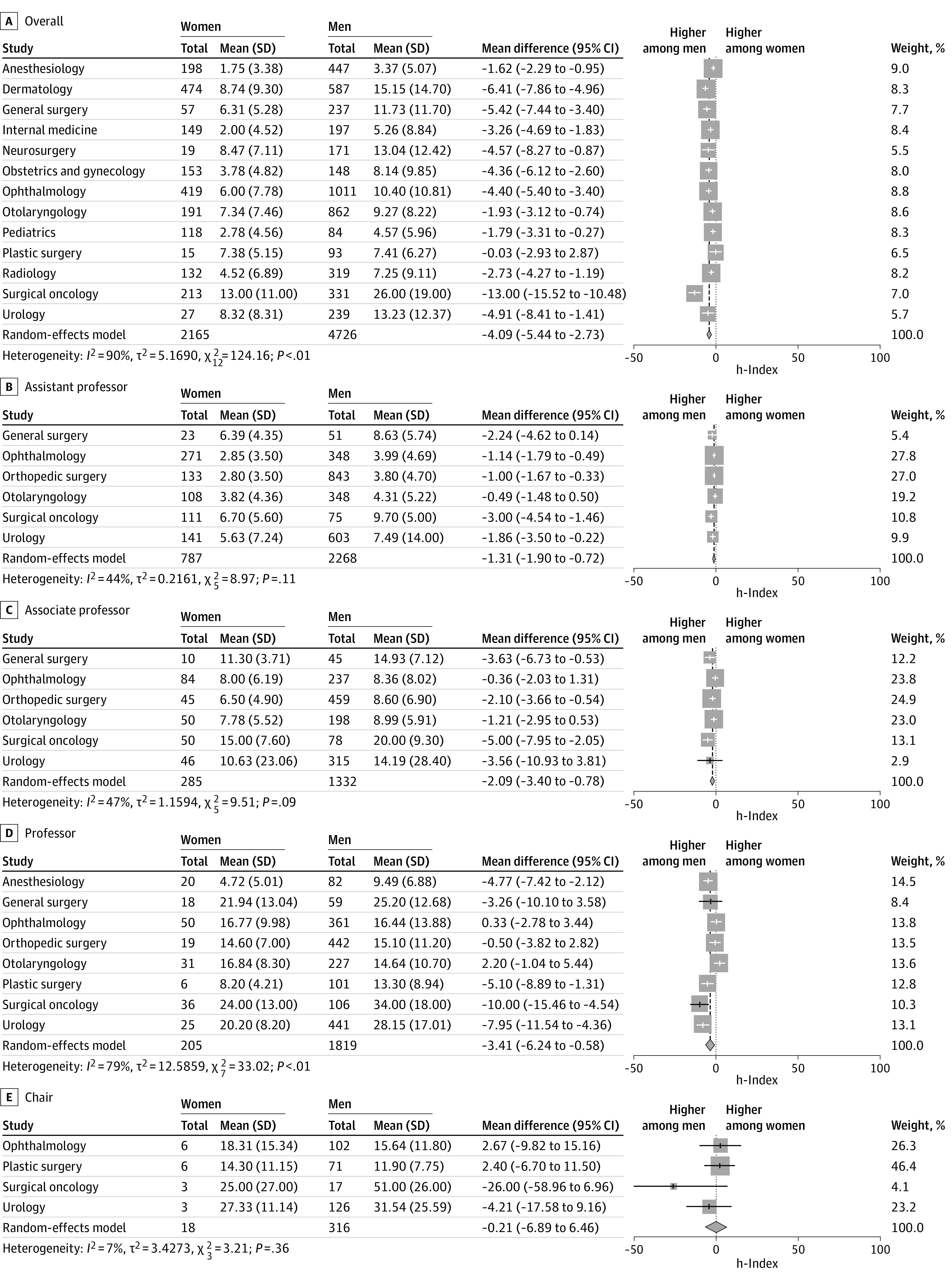
Mean Difference Between h-Indexes of Female and Male Faculty With 95% CIs, Organized by Academic Rank and Specialty Squares indicate each study’s effect size (at the center) and weight in overall analysis (box size); diamonds, overall or summary effect size; and horizontal lines, 95% CI.

eFigure 1 in the Supplement presents the mean h-indexes of female and male faculty, and Figure 2A presents the mean difference between female and male faculty. The summary effect sizes revealed the following: mean h-index for women of 6.07 (95% CI, 4.37-7.77; n = 2150), mean h-index for men of 10.32 (95% CI, 7.63-12.80; n = 5352), and mean h-index difference of −4.09 (95% CI, −5.44 to −2.73; *P* < .001). On the basis of the aforementioned mean difference and *P* value, female faculty overall have a significantly lower mean h-index than men. Mean h-indexes of female faculty were also lower than mean h-indexes of male faculty across all specialties except plastic surgery (7.38 [5.15] for female faculty vs 7.41 [6.27] for male faculty for overall plastic surgery) (Figure 2A).

eFigure 2A and B in the Supplement presents the mean h-indexes of female and male assistant professors, and Figure 2B presents the mean difference between female and male assistant professors. The summary effect sizes revealed the following: mean h-index for women of 4.58 (95% CI, 3.33-5.83; n = 787), mean h-index for men of 6.19 (95% CI, 4.73-7.66; n = 2268), and mean h-index difference of −1.31 (95% CI, −1.90 to −0.72; *P* < .001). On the basis of the aforementioned mean difference and *P* value, the mean h-indexes of female assistant professors were lower than the mean h-indexes of male assistant professors. The mean h-indexes of female faculty were also lower than the mean h-indexes of male faculty across all specialties except otolaryngology (3.82 [4.36] for female faculty vs 4.31 [5.22] for male faculty for overall otolaryngology) (Figure 2B). eFigure 2C and D in the Supplement presents the mean h-indexes of female and male associate professors, and Figure 2C presents the mean difference between female and male associate professors. The summary effect sizes revealed the following: mean h-index for women of 9.70 (95% CI, 7.20-12.20; n = 285), mean h-index for men of 12.31 (95% CI, 9.69-14.92; n = 1332), and mean h-index difference of −2.09 (95% CI, −3.40 to −0.78; *P* = .002). On the basis of the aforementioned mean difference and *P* value, the mean h-indexes of female associate professors were lower than the mean h-indexes of male professors. The mean h-indexes of female faculty were also lower than the mean h-indexes of male faculty across all specialties except ophthalmology (8.00 [6.19] for female faculty vs 8.36 [8.02] for male faculty for overall ophthalmology) and otolaryngology (7.78 [5.52] for female faculty vs 8.99 [5.91] for male faculty).

eFigure 3A and B in the Supplement presents the mean h-indexes of female and male professors, and Figure 2D presents the mean difference between female and male professors. The summary effect sizes revealed the following: mean h-index for women of 15.74 (95% CI, 10.88-20.60; n = 185), mean h-index for men of 19.40 (95% CI, 14.85-23.95; n = 1738), and mean h-index difference of −3.41 (95% CI, −6.24 to −0.58; *P* = .02). On the basis of the aforementioned mean difference and *P* value, the mean h-indexes of female professors were lower than the mean h-indexes of male professors. The mean h-indexes of female professors were also lower than the mean h-indexes of male professors across all specialties, but not always with significance (Figure 2D).

**Figure 3.**
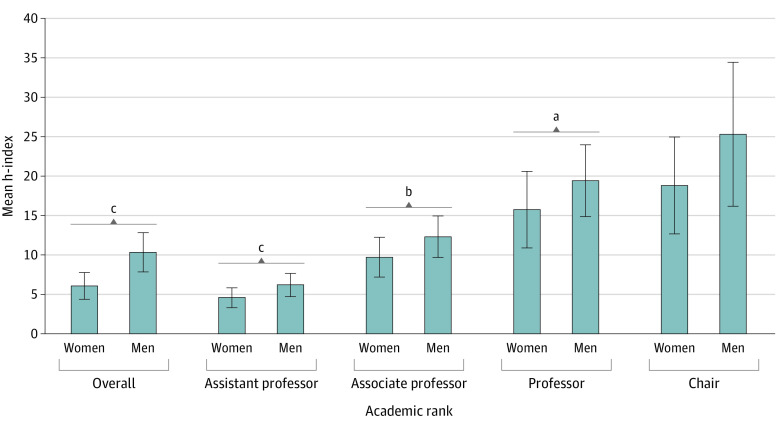
Overall Mean h-Index of Female and Male Faculty Physicians, Stratified by Rank Error bars represent 95% CIs. ^a^*P* < .05. ^b^*P* < .01. ^c^*P* < .001.

eFigure 3C and D in the Supplement presents the mean h-indexes of female and male chairs, and Figure 2E presents the mean difference between female and male chairs. The summary effect sizes revealed the following: mean h-index for women of 18.82 (95% CI, 12.68-24.96; n = 18), mean h-index for men of 25.32 (95% CI, 16.19-34.45; n = 316), and mean h-index difference of −0.21 (95% CI, −6.89 to 6.46; *P* = .95). Overall and by specialty, no significance is seen in the mean difference of the h-indexes of female and male chairs (Figure 2E). Figure 3 summarizes the overall mean h-indexes of female and male faculty physicians stratified by rank.

Table 2 presents mean and median m-indexes for various specialties, categorized by sex and academic rank. The mean m-indexes of men were generally higher than the m-indexes of women, which is consistent with the trend seen in h-indexes (0.58 vs 0.47 for radiation oncology overall, 0.6 vs 0.5 for urology overall, and 0.72 vs 0.64 for neurosurgery overall (Table 2). However, not enough data were given to be able to test for significance.

**Table 2.** Mean and Median m-Indexes by Specialty, Position, and Sex, 2009-2018

Specialty and rank	Mean m-index		Publication	Discusses possible causes?	Discusses intervention?
	Women	Men			
Radiation oncology					
Assistant professor	0.43 (n = 137)^a^	0.43 (n = 274)^a^	Holliday et al,^4^ 2014	No	No
Associate professor	0.7 (n = 54)^a^	0.54 (n = 115)^a^			
Professor or chair	0.74 (n = 34)^a^	1 (n = 211) ^a^			
Other	0.29 (n = 68)^a^	0.36 (n = 138) ^a^			
Overall	0.47 (n = 293)^a^	0.58 (n = 738) ^a^		Yes; fewer women in higher academic ranks	
Urology					
Instructor	0.14 (n = 21) ^a^	0.19 (n = 201) ^a^	Mayer et al,^39^ 2017	No	No
Assistant professor	0.46 (n = 141) ^a^	0.43 (n = 603) ^a^			
Associate professor	0.65 (n = 46) ^a^	0.68 (n = 315) ^a^			
Chair or division chief	1.19 (n = 3) ^a^	0.97 (n = 126) ^a^			
Professor	0.79 (n = 25) ^a^	0.88 (n = 441) ^a^			
Overall	0.5 (n = 236) ^a^	0.6 (n = 1686) ^a^		Yes; larger proportion of men at senior-level positions than women; longer career duration for men; women are more likely to pursue clinical-educator track; women are pigeonholed (relegated to less academically productive subspecialties); women have more familial responsibilities than men	Yes; more same-sex mentors and better opposite-sex mentors to provide better mentorship for female urologists
Neurosurgery overall	0.64 (n = 81)	0.72 (n = 1144)	Khan et al,^31^ 2014	Yes; women produce fewer but more significant impact publications	No

^a^ Median instead of mean.

## Discussion

This is the first published systematic review and meta-analysis, to our knowledge, that analyzed sex differences across multiple specialties while adjusting for academic rank, although many single-specialty studies^4,20,24,29,30,31,32,33,34,35,36,37,38,39,40^ have focused on sex differences in academic productivity. The results suggest that female faculty have lower h-indexes (−4.09; 95% CI, −5.44 to −2.73; *P* < .001) than male faculty (Figure 1, Figure 2, and Table 1). By academic rank, women have lower h-indexes in the ranks of assistant professor (−1.3; 95% CI, −1.90 to −0.72; *P* < .001), associate professor (−2.09; 95% CI, −3.40 to −0.78; *P* = .002), and professor (−3.41; 95% CI, −6.24 to −0.58; *P* = .02) (Figure 2, Figure 3, and Table 1). These results highlight the pervasive sex disparities that exist in citation-related publication productivity metrics within academic medicine. Although observed sex differences are not seen across all specialties,^46^ these data illuminate the need for ongoing discussion of the potential contributing factors to the observed sex differences to ensure equitable engagement of the full pipeline of available contributors to the field because the causes for these differences are still unclear.

Several modifiable institutional and cultural factors may contribute to these observed sex differences.^4,24,29,30,31,32,33,34,35,36,37,38,39,40^ A lack of women in senior positions may limit visible role models, contributing to a culture that encourages bias.^47^ This issue is particularly germane to women in specialties that are historically male dominated.^48^ Leaders may unconsciously select protégés who look like them with regard to sex and race, and this homophily can propagate current disparities.^49^ Therefore, women may not have equal access to high-quality mentors and sponsors, valuable networking and research collaboration, or leadership opportunities, as exemplified by the fewer women seen in higher leadership roles^7,8,50,51^ and the tendency of women to be on practitioner-educator rather than tenure tracks.^52^ Studies^53,54^ in STEM (science, technology, engineering, and math) fields have found that women have fewer research opportunities early in their careers, which may have negative effects on future academic impact. Women in academic medicine receive less initial start-up and subsequent research funding than men.^13,14^ Even at senior levels, they are less likely to hold endowed professorships.^55^ Therefore, women have fewer opportunities, resources, and protected time to be able to conduct high-quality research.^19,24,56^

Implicit bias and the tendency of networks to exclude women also reduce their likelihood to be invited to speak in prominent venues, write author-invited editorials, or participate in other activities that increase the visibility and subsequent citation of the work they produce.^11,57^ Journal peer review processes may be biased, given the typical reliance on single-blinded peer review, and studies^58,59^ have even documented differences in the descriptive language used by men and women that may influence numbers of citations. Men are also more likely to engage in self-promotion, and self-promotion by men is less likely to violate societal norms.^60,61,62,63,64,65,66^ Both traditional news media and novel forms of calling attention to published work, such as Twitter, also amplify men’s voices more than women’s.^67,68^

Sex differences in home and caretaking responsibilities may also play a role. Evidence suggests that sex differences in career trajectories in academic medicine are not simply a reflection of differences in women’s priorities.^69^ Structural challenges persist, such as the collision between “the biological clock” and “the tenure clock” and continued expectations of society for a sex-based division of domestic labor. Although involvement by male parents has increased during recent decades, there are unavoidable differences in the impact of pregnancy, childbirth, and breastfeeding on female physicians during training and/or early in their careers,^70^ and women in academic medicine are disproportionately responsible for caretaking and other domestic responsibilities.^28,71^ Women with children receive less institutional support and publish less than men with children.^27^ Such issues are likely amplified in the current environment of school closures and social distancing caused by the COVID-19 pandemic.^72,73^

Sexual harassment and sex discrimination also demonstrably impact women’s career choices and academic success.^74,75^ Women who seek advancement are more vulnerable to sexual harassment than men, and this risk may deter women from pursuing academic opportunities.^76^ Because sexual harassment is more common in fields where women do not share equally in power and authority, a vicious cycle exists whereby its eradication depends on efforts to ensure that women are fully integrated at all levels.

Pointing out observed sex differences does little good unless the numbers are accompanied by productive discussion and suggestions for improvement. Several of the above-referenced studies^4,24,30,31,33,36,37,38,39^ mentioned contributing factors that are modifiable with intervention. The #MeToo movement prompted interest in establishing policies and programs that make the workplace safer for women physicians.^77,78,79^ Some (but not all) studies^80,81^ have found that implicit bias training is helpful in breaking ingrained sex bias habits to support career advancement for women in science, technology, engineering, and math fields. Formal mentorship programs also have the potential to improve the academic footprint of female physicians, particularly in their early careers.^82,83,84^ Increased mentorship, however, is not enough, as seen by the dearth of women in upper faculty ranks to this day.^7^ With an established association between sponsorship and academic success, increased sponsorship is also needed to improve the academic influence of female physicians.^50,85^ Nationally recognized career development programs can also retain women in the academic pipeline and help women prepare for leadership roles.^75,86,87,88^ Interventions that serve to promote the academic work of women are also important to in turn increase the academic influence of women.^60,61,62,63,64,65,66^ Finally, institutional policies to promote work-life integration, such as programs and grants that target individuals with substantial extraprofessional caregiving responsibilities, have the potential to ensure all faculty have the opportunity to contribute their insights.^88,89,90^

The reasons that the included studies^24,30,33,36,37,39^ cited to explain the sex gap, such as lack of effective mentorship for women and the disparate influence of familial responsibilities on women, aligned with the reasons that we discussed previously. Another proposed reason was the tendency for women to have more clinical and educational responsibilities than men, which takes away from their time to do academic work.^24,33,36,38,39,52^ Only 3 studies^33,34,39^ listed proposed interventions. The interventions focused on increasing effective mentorship for women, emphasizing that women perform research early, and recruiting women who are research-minded early as residents or medical students to pursue an academic career. We believe that this synthesis of the broader literature helps to highlight a number of other interventions that should also be evaluated.

### Limitations

This study has limitations. Many specialties were excluded because of the dearth of studies looking at sex differences. Furthermore, many included specialties are male dominated; therefore, the percentage of women in this meta-analysis is lower than the overall representation of women in academic medicine, limiting the generalizability of the findings. In addition, the need to approximate some data and the use of multiple databases slightly diminish the findings’ accuracy.^24,39^ In addition, a meta-analysis was performed instead of an analysis of a random sample of faculty at academic medical institutions because data on individual faculty were lacking. The h-index and m-index have their own limitations as citation-related publication productivity metrics because they do not account for journal impact or author placement, are prone to inflation by coauthorship and self-citations, and do not fully account for time.

## Conclusions

The results of this study suggest that the h-indexes of women are lower than those of men across medical specialties and academic ranks, corroborating the sex differences described in many previous single-specialty studies^4,24,29,30,31,32,33,34,35,36,37,38,39,40^ and suggesting that more pervasive sex differences exist across academic medicine. This study provides important benchmarking information and motivation for further investigation of potential causes of these observed sex differences and mitigation of modifiable factors that influence them.

## Notes

Supplement. **eTable.** PICOS Inclusion Criteria for Population, Intervention, Control, Outcome, and Study **eFigure 1.** Mean h-Indexes and 95% Confidence Interval of Male and Female Medical Faculty, Organized by Academic Specialty **eFigure 2.** Mean h-Indexes and 95% Confidence Interval of Assistant Professors and Associate Professors, Organized by Gender and Academic Specialty **eFigure 3.** Comparison of Mean h-Indexes and 95% Confidence Interval of Professors and Chairs, Organized by Academic Specialty
