# Supplementary material for: Sex Differences in Academic Productivity Across Academic Ranks and Specialties in Academic Medicine: A Systematic Review and Meta-analysis
Source: JAMA Netw Open. 2021 Jun 29;4(6):e2112404. doi: 10.1001/jamanetworkopen.2021.12404 (PMC8243235; doi:10.1001/jamanetworkopen.2021.12404)
Supplement: Supplement. — eTable. PICOS Inclusion Criteria for Population, Intervention, Control, Outcome, and Study eFigure 1. Mean h-Indexes and 95% Confidence Interval of Male and Female Medical Faculty, Organized by Academic Specialty eFigure 2. Mean h-Indexes and 95% Confidence Interval of Assistant Professors and Associate Professors, Organized by Gender and Academic Specialty eFigure 3. Comparison of Mean h-Indexes and 95% Confidence Interval of Professors and Chairs, Organized by Academic Specialty [file jamanetwopen-e2112404-s001.pdf]

Supplementary Online Content

Ha GL, Lehrer EJ, Wang M, Holliday E, Jagsi R, Zaorsky NG. Sex differences in academic productivity across academic ranks and specialties in academic medicine: a systematic review and meta-analysis. *JAMA Netw Open*. 2021;4(6):e2112404. doi:10.1001/jamanetworkopen.2021.12404

eTable. PICOS Inclusion Criteria for Population, Intervention, Control, Outcome, and Study

eFigure 1. Mean h-Indexes and 95% Confidence Interval of Male and Female Medical Faculty, Organized by Academic Specialty

eFigure 2. Mean h-Indexes and 95% Confidence Interval of Assistant Professors and Associate Professors, Organized by Gender and Academic Specialty

eFigure 3. Comparison of Mean h-Indexes and 95% Confidence Interval of Professors and Chairs, Organized by Academic Specialty

This supplementary material has been provided by the authors to give readers additional information about their work.

© 2021 Ha GL et al. *JAMA Network Open.*

eTable. PICOS inclusion criteria for Population, Intervention, Control, Outcome, and Study

**Population**	Faculty in academic medicine with reported h-index, stratified by gender.
**Intervention**	None. Study must report h-index.
**Control**	N/A
**Outcomes**	H-index, either as mean or median. Other publication metrics if available, including number of citations, number of publications, m-index.
**Study Design**	Observational studies.

© 2021 Ha GL et al. *JAMA Network Open.*

eFigure 1. Mean h-indexes and 95% confidence interval of male and female medical faculty, organized by academic specialty

(a) Overall - Women

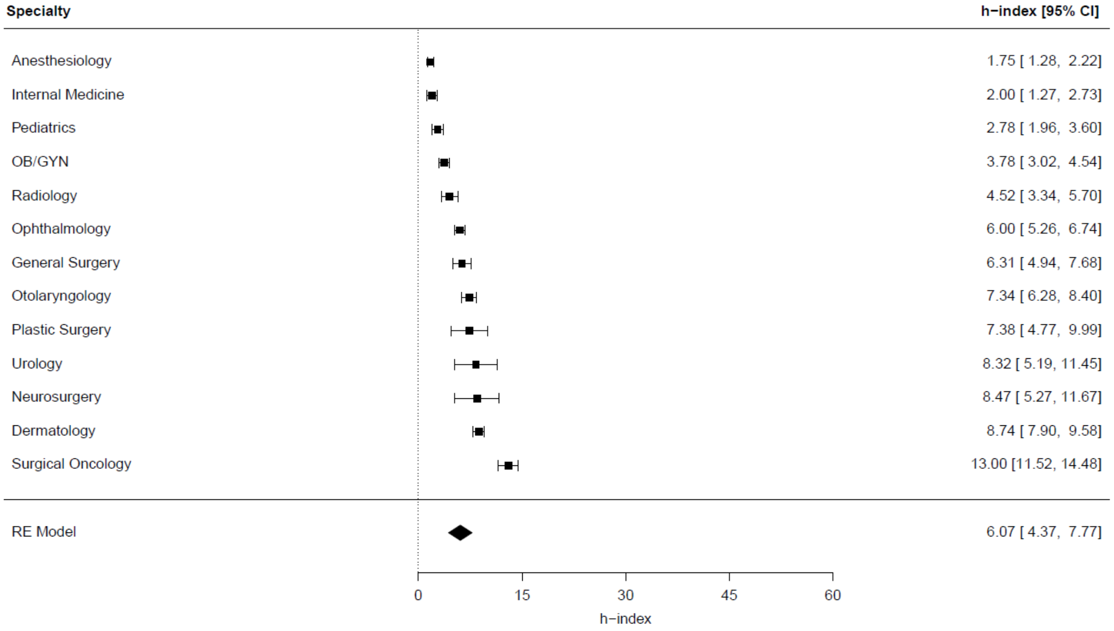

(b) Overall - Men

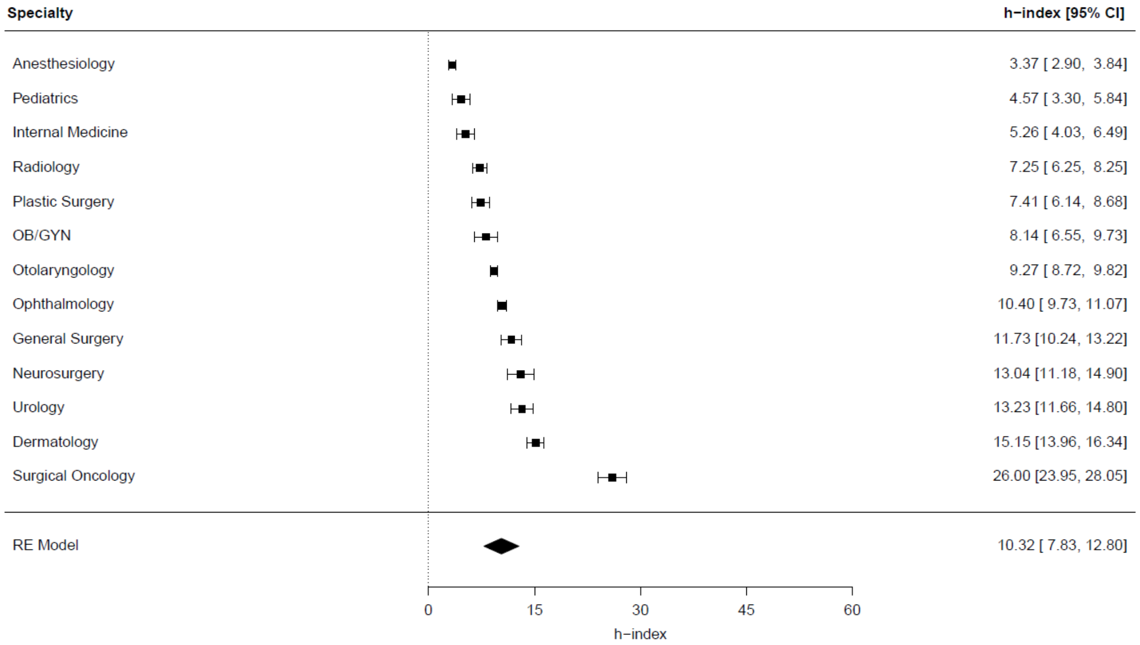

The two forest plots depict mean h-indexes of women and men categorized by academic medical specialty. The top pane (Supplemental Figure 1a) displays the mean h-indexes for women, and

© 2021 Ha GL et al. *JAMA Network Open.*

the bottom pane (Supplemental Figure 1b) displays the mean h-indexes for men. Based on summary effect size under random effects model, the mean h-index for each medical specialty at each rank is listed, with 95% confidence intervals (CIs) in brackets. The black square represents the individual study's effect. The size of the square varies to reflect the weight a particular study has in the overall analysis. The black line represents the CI of a study. The diamond represents the overall mean h-index with 95% confidence interval.

© 2021 Ha GL et al. *JAMA Network Open.*

eFigure 2. Mean h-indexes and 95% confidence interval of assistant professors and associate professors, organized by gender and academic specialty

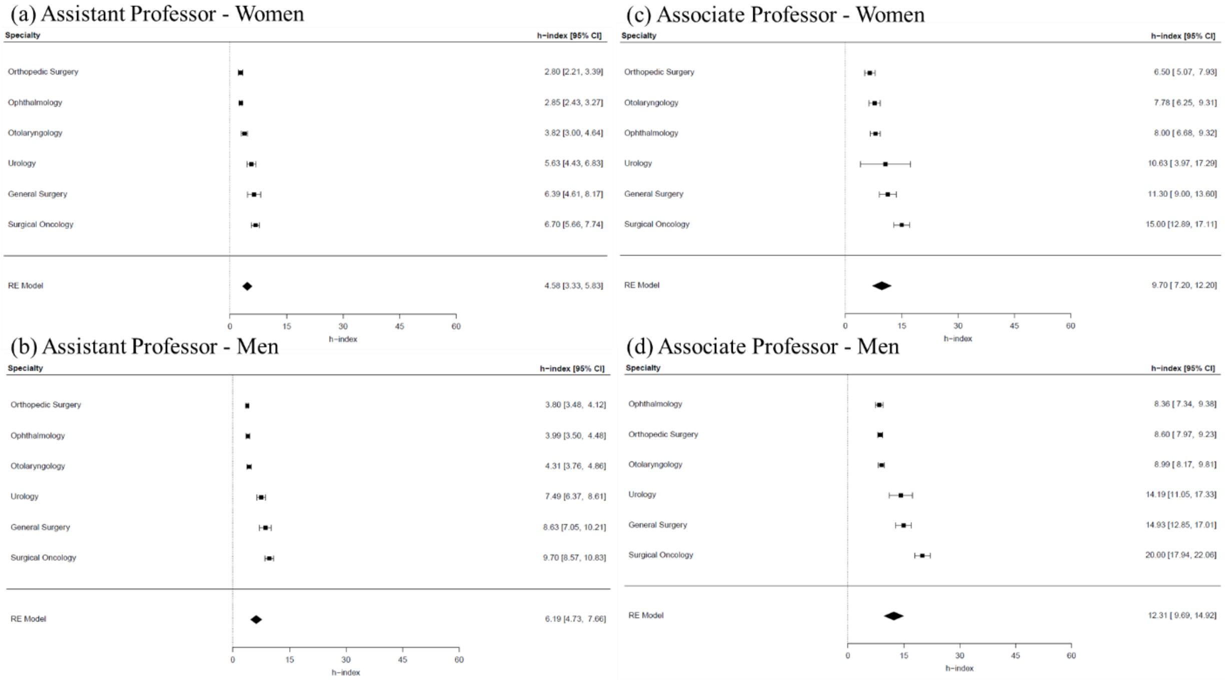

Legend:

The left two forest plots depict mean h-indexes of female and male assistant professors, categorized by academic medical specialty. The top pane (eFigure 3a) displays the mean h-indexes for women, and the bottom pane (eFigure 3b) displays the mean h-indexes for men. The right two forest plots depict mean h-indexes of female and male associate professors, categorized by academic medical specialty. The top pane (eFigure 2c) displays the mean h-indexes for women, and the bottom pane (eFigure 2d) displays the mean h-indexes for men. Based on summary effect size under random effects model, the mean h-index for each medical specialty at each rank is listed, with 95% CIs in brackets. The black square represents the individual study's effect. The size of the square varies to reflect the weight a particular study has in the overall analysis. The black line represents the CI of a study. The diamond represents the overall mean h-index with 95% confidence interval.

© 2021 Ha GL et al. *JAMA Network Open.*

eFigure 3. Comparison of mean h-indexes and 95% confidence interval of professors and chairs, organized by academic specialty

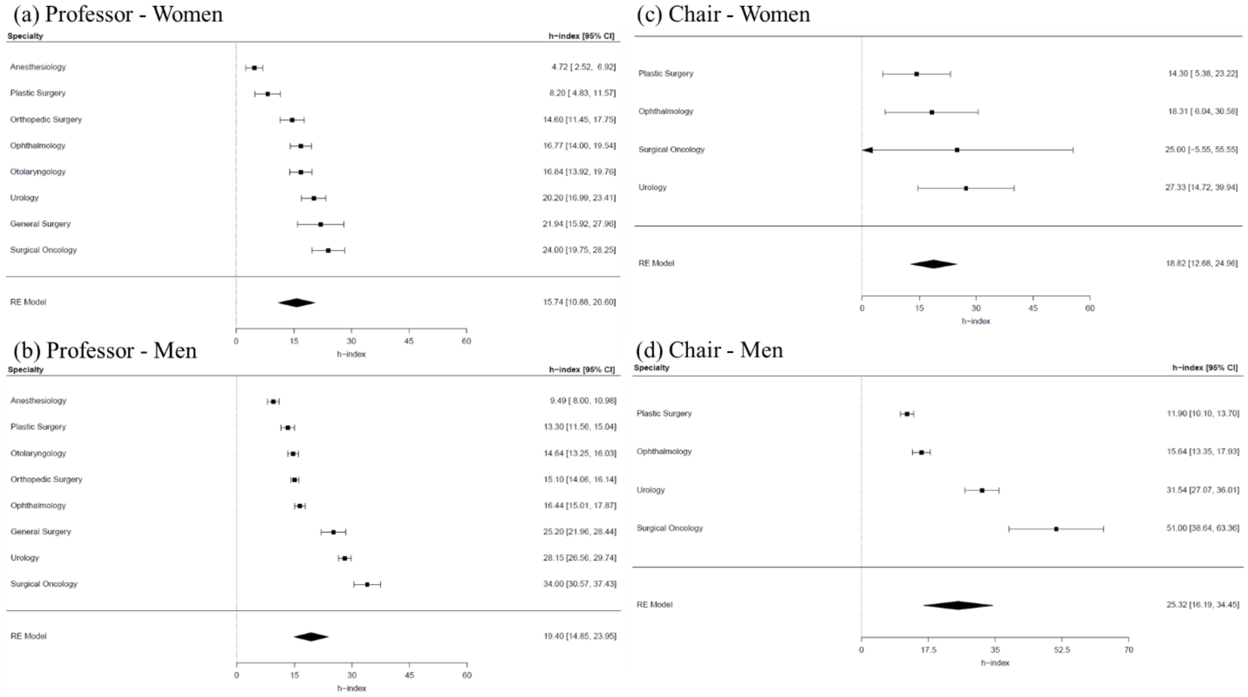

Legend:

The left two forest plots depict mean h-indexes of female and male professors, categorized by academic medical specialty. The top pane (eFigure 3a) displays the mean h-indexes for women, and the bottom pane (eFigure 3b) displays the mean h-indexes for men. The right two forest plots depict mean h-indexes of female and male chairs, categorized by academic medical specialty. The top pane (eFigure 3c) displays the mean h-indexes for women, and the bottom pane (eFigure 3d) displays the mean h-indexes for men. Based on summary effect size under random effects model, the mean h-index for each medical specialty at each rank is listed, with 95% CIs in brackets. The black square represents the individual study's effect. The size of the square varies to reflect the weight a particular study has in the overall analysis. The black line represents the

© 2021 Ha GL et al. *JAMA Network Open.*

CI of a study. The diamond represents the overall mean h-index effect with 95% confidence interval.

© 2021 Ha GL et al. *JAMA Network Open.*
